# Medical Convention

**Published:** 1847-07

**Authors:** Ro. W. Haxall

**Affiliations:** Chairman


*Medical Convention in Philadelphia.—Report on a Uniform and Elevated Standard of Requirements for the Degree of M.D.* We find the subjoined report on this subject in the Buffalo Medical Journal, and hasten to lay it before our readers:

In order to obtain a knowledge of the requirements demanded by the various schools of the country, a letter was addressed to all whose existence was known to your committee, requesting them to furnish the desired information; these consisted of thirty-three in number, and answers were received from nineteen, being a fraction more than one-half. In the majority of instances pamphlets were forwarded to the chairman, containing the rules and regulations of the colleges, together with the branches taught; while from others letters were received, simply stating that two full courses of lectures were demanded, that the candidate must be twenty-one years of age, of good moral character, etc. From this correspondence but little available information was derived. A circular was then dispatched to the different colleges, propounding the following questions, answers to which were respectfully solicited. Nineteen of these circulars were filled up and returned. State—

1. The number of students during the session of 1845, '6;
2. The number of graduates in 1845, '6;
3. The number of charity pupils in 1845, '6;
4. The number of professors attached to your school;
5. The date at which lectures begin and end;
6. The requirements for a degree;
7. Is the inquiry made, previous to examination, whether cr not these requirements are fulfilled ?
8. Is any evidence of having attended a course of clinical instruction necessary to graduation ?
9. Is it required of crndidates for graduation that they shall have devoted any time to dissection ?

The responses to these several questions have been arranged in tabular form, from which the result of the whole correspondence may be ascertained at a glance, and which your committee beg leave to lay before this convention as a part of this report. We cannot avoid the expression of our regret that so many of the colleges have failed to respond to our circular—not, we hope, from a listless indifference to the subject, or a determination not to abide by the recommendations of this body. Information was also requested from the proper authorities in regard to requirements of surgeons in the army and navy of the United States; a reply was received and is appended in a note.

In summing up the information obtained from the nineteen colleges named in the table, in the order in which the questions were proposed, it will be found that the number of students belonging to these institutions during the session of 1845, '6, amounted to 2544, including 31 nonpaying pupils; and that the number of graduates was 730. The number of professors varies from three to eight. It may be proper to remark that the lowest number here named is attached to the University of Virginia, where the usual branches of medical education are taught, and where the term of study extends throughout the period of nine months. If we exclude this institution from our table, the minimum is five and the maximum is eight. The time employed in lecturing also varies, thirteen weeks being the shortest and eighteen the longest period; sixteen weeks, however, are devoted to the lectures in a large majority of the schools. The requirements for the degree of M.D. appear to be very general: the candidate must be twenty-one years of age, his moral character good, his examination satisfactory, his thesis passable, and he must have attended two full courses of lectures to be included within the period of three years' study. In some schools a practice of four years and an attendance on one course of lectures is sufficient to entitle the individual to an examination. Branches are taught in some institutions which are omitted in others, and the manner in which these are distributed is by no means uniform. It appears without exception that the inquiry is made previous to examination whether or not all the requirements have been fulfilled, and in some cases unquestionable proof of the fact must be adduced. Evidence of having attended a course of clinical instruction is required in twelve of the colleges, while in seven it is not; and as to dissection, five render it obligatory, and the remaining fourteen are content to urge its recommendation.

Such, then, is the information which the committee, after diligent inquiry, is enabled to lay before this body. It would have been a matter of some importance to have received answers from all the schools, for in that case an account entirely accurate of the whole number of students in the United States would have been obtained. It will be seen that the nineteen colleges which noticed our circular report the aggregate number to be 2544; and if it be proper to assign the same ratio to the fourteen which did not, the whole number of students during the session of 1845, '6, will be found to be 4418 and a fraction. We believe this to be a very fair statement, for several statistical tables, published from time to time in the journals, have varied but little from this amount; and if we extend the same rule to the classes of graduates, we reach the conclusion that a fraction less than 1300 were added to the already existing number of physicians, in the spring of 1846.

The very large number of physicians in the United States—

a number far larger in proportion to its population than in any country, perhaps, of which we have a correct knowledge—has frequently been the subject of remark. To relieve the diseases of something more than twenty millions of people, we have an army of doctors amounting, by a recent computation, to forty thousand, which allows one to about every five hundred inhabitants. And if we add to the forty thousand the long list of irregular practitioners who swarm like locusts in every part of the country, the proportion of patients will be still further reduced. No wonder, then, that the profession of medicine has measurably ceased to occupy the elevated position which it once did; no wonder that the merest pittance in the way of remuneration is scantily doled out even to the most industrious in our ranks; and no wonder that the intention, at one time correct and honest, will occasionally succumb to the cravings of a hard necessity. The evil must be corrected. With a government like ours, to diminish the number of medical schools is not to be expected; and the corrective can alone be found in the adoption of such a standard of requirements—in the general estimate of which the recommendations of this committee will form but an item—as will place the diploma beyond the reach of those who seek to wear its honors without deserving them.

The shortness of the time allotted to the delivery of lectures we believe to be an evil of no small magnitude. It is next to an impossibility that the strongest intellect can receive and well digest some half a dozen discourses or more a day, embracing subjects which have oftentimes little or no immediate connection with each other. The mind becomes wearied with the multiplicity of its occupations, and the thoughts of today are forgotten in the constantly recurring and harassing duties of the morrow. A proper allotment of time cannot be given to that deep reflection which the importance of the subject demands, and without which no solid advancement can be made. There are others, too, who do not always rest satisfied with professorial prelections, who approve in its fullest extent the adage, "Nullius addictus jurare in verba magistri," and who seek in the recorded experience of others the widest extent of information. Nor can teachers themselves, with every disposition to impart the fullest instruction, do justice to their several branches within the limited period of four months. For this reason the examination, which may indeed test the actual acquirement of the student, can never embrace an extensive field of knowledge. Under the present regulation this cannot be expected, for justice to the pupil must prevent an inquiry into subjects in which he has never been instructed.

In regard to the branches to be taught, it is not improbable that the recommendations of the committee may not meet the views of some of the members of this convention. It may be thought that others should have been added, and that consequently the number of professors should have been considerably augmented. This subject was freely canvassed by the committee, and the opinion was general that in the commencement of our effort to reform the existing condition of things, too high a standard of requirement ought not to be immediately insisted on. The conclusions to which this convention may come upon any subject cannot be forced upon the schools contrary to their wishes. In the advice of this body there exists, however, a moral power which it would be unwise to disregard—a power which, while it seeks to elevate medical requirement, should not impose a single requisition which is not entirely practicable. Besides, it is to be recollected that the decrees of this convention need not be final and unalterable. The National Medical Association, contemplated under the first two resolutions adopted at the meeting in May last, which it is to be hoped will convene from time to time in different parts of the country, can legitimately exercise the same influence which we now seek to do; and when in after time it may be deemed advisable to add new subjects of instruction, it will not hesitate to express its opinion. Under this view of the matter, your committee advise that lectures be given on the following branches in all the colleges, viz: On the theory and practice of medicine; the principles and practice of surgery; general and special anatomy; physiology and pathology; materia medica; therapeutics and pharmacy; midwifery, and diseases of women and children; chemistry and medical jurisprudence. These subjects, it is true, are now taught in the majority of our schools. With a lengthened period for teaching, a double advantage will be gained: a wider extent of information may be imparted to the student, while his time will be occupied with fewer lectures during the day. As to the appropriation of the several branches, it is deemed inexpedient to make any suggestion, as the circumstances of each institution may be such as will necessarily lead to a different arrangement in this particular. It is believed, however, that a slight augmentation in the number of professors will be necessary.

In regard to clinical instruction, your committee would willingly have made it a requisition to the attainment of the diploma; but an insuperable difficulty seemed to exist: it is believed that few of the colleges have hospitals attached to them, and in very many instances where such institutions exist in the cities, the professors do not receive the appointment of attending physicians and surgeons. Consequently the opportunity is lost to them of imparting such instruction as would be valuable to the student. We cannot, however, close this portion of our report without urging the very great importance of clinical instruction. Many a young man has entered upon the duties of his profession, sufficiently instructed, it may be, in its principles, who has most painfully felt the responsibility of his position, in default of a certain degree of practical knowledge; indecision must often fetter his efforts, to the detriment of his patient, and that selfconfidence which can alone restore serenity to his mind must be purchased by a long period of perplexing doubt. During the pupilage of the student, he should lose no opportunity of witnessing cases of disease wherever they are to be found. Instructors themselves should esteem it their highest duty to introduce their pupils to the bedside of their patients in all cases where propriety will admit of it. The hospitals, penitentiaries and poor-houses, found in so many of our towns and cities, will furnish from time to time cases of every description, while the pauper practice of the country will prove no indifferent means of imparting clinical information. Instruction gained from these sources in free and familiar conversation with the preceptor will perhaps be of more avail than any other; for the large number of students who occasionally throng the wards of a hospital render it impossible for each one to obtain a correct knowledge of the cases presented to him. We do not intend by this remark to convey the idea that information given in this way would be valueless. On the contrary, when properly conducted it may made productive of the greatest good. Let the professor daily analyze the symptoms which arise in every case, have them carefully noted down in his ward-book, together with his prescription, and at its termination let him review the whole ground, aided by a postmortem examination if it result unfavorably, and he will impart instruction which is truly valuable.

It will be seen that only five of the colleges insist upon dissection as a requisite to graduation. Your committee accord in the opinion, that all should render it obligatory upon the student to devote some portion of his time to this needful pursuit. To enter into an argument to prove its absolute necessity, not only to the surgeon, but also to the physician, would be a work of supererogation.

Ro. W. Haxall, Chairman.

